# Pathologic complete response after neoadjuvant systemic therapy for breast cancer in *BRCA* mutation carriers and noncarriers

**DOI:** 10.1038/s41523-024-00674-y

**Published:** 2024-07-26

**Authors:** Sara P. Myers, Varadan Sevilimedu, Andrea V. Barrio, Audree B. Tadros, Anita Mamtani, Mark E. Robson, Monica Morrow, Minna K. Lee

**Affiliations:** 1 Division of Breast Surgery, Department of Surgery, Brigham and Women’s Hospital, Boston, MA USA; 2 Breast Oncology Program, Dana-Farber Brigham Cancer Center, Boston, MA USA; 3 Harvard Medical School, Boston, MA USA; 4 Biostatistical Service, Department of Epidemiology and Biostatistics, Memorial Sloan Kettering Cancer Center, New York, NY USA; 5 Breast Service, Department of Surgery, Memorial Sloan Kettering Cancer Center, New York, NY USA; 6 Breast Medicine Service, Department of Medicine, Memorial Sloan Kettering Cancer Center, New York, NY USA

**Keywords:** Breast cancer, Surgical oncology

## Abstract

*BRCA1 and BRCA2* pathogenic variant carriers develop breast cancers with distinct pathological characteristics and mutational signatures that may result in differential response to chemotherapy. We compared rates of pathologic complete response (pCR) after NAC between *BRCA1/2* variant carriers and noncarriers in a cohort of 1426 women (92 [6.5%] *BRCA1* and 73 [5.1%] *BRCA2*) with clinical stage I–III breast cancer treated with NAC followed by surgery from 11/2013 to 01/2022 at Memorial Sloan Kettering Cancer Center. The majority received doxorubicin/cyclophosphamide/paclitaxel therapy (93%); *BRCA1/2* carriers were more likely to receive carboplatin (*p* < 0.001). Overall, pCR was achieved in 42% of *BRCA1* carriers, 21% of *BRCA2* carriers, and 26% of noncarriers (*p* = 0.001). Among clinically node-positive (cN+) patients, nodal pCR was more frequent in *BRCA1/2* carriers compared to noncarriers (53/96 [55%] vs. 371/856 [43%], *p* = 0.015). This difference was seen in HR+/HER2− (36% vs. 20% of noncarriers; *p* = 0.027) and TN subtypes (79% vs. 45% of noncarriers; *p* < 0.001). In a multivariable analysis of the overall cohort, *BRCA1* status, and TN and HER2+ subtypes were independently associated with pCR. These data indicate that *BRCA1* carriers may be more likely to achieve overall and nodal pCR in response to NAC compared with *BRCA2* carriers and patients with sporadic disease. Further studies with a larger cohort of *BRCA1/2* mutation carriers are needed, as a small sample size may have a restricted ability to detect a significant association between mutational status and pCR in sensitivity analyses stratified by subtype and adjusted for clinically relevant factors.

## Introduction

*BRCA1* and *BRCA2* are the most commonly mutated cancer susceptibility genes in hereditary breast cancers^1^; affected carriers have a 2–3% annual risk of developing disease^2^. The epidemiology of breast cancers arising in the context of a *BRCA1 or BRCA2* mutation is different than that of sporadic cancers. These cancers tend to be higher grade and, in the case of *BRCA1* mutations, are more likely to be triple negative^3^. Whether this variability leads to differences in response to systemic therapy between carriers and noncarriers remains unclear^4–8^.

Certain functions of *BRCA1* and *BRCA2* proteins, such as their role in DNA repair, directly impact cellular response to chemotherapeutic agents^9,10^. Additionally, tumors associated with *BRCA1/2* mutations display distinct mutational signatures and gene expression profiles^3^. These factors may affect response to the standard chemotherapy regimen traditionally given to patients with sporadic breast cancers^11–13^.

In the case of neoadjuvant chemotherapy (NAC), patients with *BRCA1* pathogenic mutations have been shown to have high pathologic complete response (pCR) rates, ranging from 24% to 90%^14–18^. Patients with *BRCA2* pathogenic mutations, conversely, seem to have similar pCR rates (13%–53%) to those with sporadic breast cancer^1,19^. However, the small sample sizes of these studies prevented investigation of differences in response to NAC between *BRCA1* vs. *BRCA2* carriers. We describe a robust single-institutional experience that addresses this gap in knowledge by distinguishing between rates of pCR after NAC in *BRCA1* compared to *BRCA2* carriers and noncarriers.

## Results

Of 1426 patients included in this study, 92 (6.5%) and 73 (5.1%) were *BRCA1* and *BRCA2* pathogenic variant carriers, respectively. Compared to noncarriers, *BRCA1* and *BRCA2* carriers were younger (*p* < 0.001), more frequently presented with clinical T1 disease (*p* = 0.002), and more frequently underwent bilateral mastectomy (*p* < 0.001). Regarding molecular subtype, 73% (67/92) of *BRCA1* carriers presented with TNBC, and 60% (44/73) of *BRCA2* carriers presented with HR+ disease. Among noncarriers who received NAC, 38% (481/1261) were HER2+ and 25% (317/1261) had TNBC. *BRCA1-*associated tumors were more frequently poorly differentiated compared to *BRCA2-*associated and sporadic cancers (*p* = 0.001). Almost all patients received doxorubicin/cyclophosphamide/paclitaxel therapy (93%); *BRCA1* carriers were more likely to receive carboplatin (*p* < 0.001) (Table 1).

**Table 1:** Patient, tumor, and treatment characteristics

Characteristic	*BRCA1* *n* = 92	*BRCA2* *n* = 73	Noncarrier *n* = 1261	*p* value
Age	37 (33–46)	42 (36–51)	52 (44–61)	<0.001
Race				0.2
Black	12 (13%)	7 (10%)	180 (16%)	
Asian-American	11 (12%)	18 (26%)	213 (18%)	
White	66 (74%)	44 (64%)	765 (66%)	
Unknown	3	4	103	
Clinical T stage				0.002
T0/Tis/T1	19 (21%)	22 (30%)	182 (14%)	
T2	53 (58%)	30 (41%)	699 (55%)	
T3	17 (18%)	16 (22%)	250 (20%)	
T4	3 (3.3%)	5 (6.8%)	130 (10%)	
Clinical *N* stage				0.011
N0	44 (48%)	25 (34%)	405 (32%)	
N1	45 (49%)	45 (62%)	743 (59%)	
N2/3	3 (3.3%)	3 (4.1%)	113 (9%)	
Differentiation				0.001
Poorly differentiated	78 (88%)	48 (67%)	873 (70%)	
Well or moderately differentiated	11 (12%)	24 (33%)	382 (30%)	
Unknown	3	1	6	
Histology				0.067
Ductal	90 (98%)	64 (89%)	1132 (91%)	
Lobular/mixed	2 (2.2%)	8 (11%)	106 (8.6%)	
Unknown	0	1	23	
Receptor subtype				<0.001
HR+/HER2−	21 (23%)	38 (52%)	463 (37%)	
HR+/HER2+	4 (4.3%)	6 (8.2%)	307 (24%)	
HR−/HER2+	0	2 (2.7%)	174 (14%)	
TN	67 (73%)	27 (37%)	317 (25%)	
ACT-based NAC	90 (98%)	71 (97%)	1153 (92%)	0.032
Carboplatin	29 (32%)	13 (18%)	140 (11%)	<0.001
Surgery				<0.001
Partial mastectomy	5 (5.4%)	3 (4.1%)	571 (46%)	
Unilateral mastectomy	3 (3.3%)	5 (6.8%)	376 (30%)	
Bilateral mastectomy	84 (91%)	65 (89%)	304 (24%)	
Unknown	0	0	10	
Axillary surgery				<0.001
SLNB	65 (71%)	47 (64%)	643 (51%)	
ALND	27 (29%)	26 (36%)	618 (49%)	

Categorical data presented as *n* (%) and continuous as median (interquartile range).

*HR* hormone receptor, *HER2* human epidermal growth factor receptor 2, *TN* triple negative, *ACT* adriamycin cyclophosphamide and taxol, *NAC* neoadjuvant chemotherapy, *SLNB* sentinel lymph node biopsy, *ALND* axillary lymph node dissection.

### Response to NAC in overall cohort

Overall pCR occurred in 42% (39/92) of *BRCA1* carriers, 21% (15/73) of *BRCA2* carriers, and 26% (327/1261) of noncarriers (*p* = 0.001). Among clinically node-positive (cN+) patients, nodal pCR was more frequent in *BRCA1* carriers (65% [31/48]) compared to *BRCA2* carriers (46% [22/48]) and noncarriers (43% [371/856]) (*p* = 0.015; Table 2). Lower cT stage (*p* = 0.028), cN0 (*p* = 0.010), *BRCA1* status (*p* = 0.001), poorly differentiated tumors (*p* < 0.001), ductal histology (*p* < 0.001), TN and HER2+ subtypes (*p* < 0.001), carboplatin receipt (*p* = 0.041), and absence of lymphovascular invasion (LVI) (*p* < 0.001), were associated with overall pCR on univariate analysis. After adjusting for differentiation and subtype, *BRCA1* carrier status remained independently associated with pCR (OR 2.37 [95% CI 1.32–4.18]; *p* = 0.003). Relative to HR+/HER2− tumors, HR+/HER2+, HR−/HER2+, and TNBC had higher odds of pCR (Table 3). Of patients with TNBC who received carboplatin, 11/24 (46%) of *BRCA1* carriers achieved pCR compared with 3/9 (33%) *BRCA2* carriers and 23/77 (30%) of noncarriers.

**Table 2:** Rates of overall and nodal pathologic complete response in *BRCA* carriers and noncarriers

	Overall pCR *n*/*N* (%)			Nodal pCR *n*/*N* (%)		
	*BRCA1*	*BRCA2*	Noncarrier	*BRCA1*	*BRCA2*	Noncarrier
All subtypes	39/92 (42%)	15/73 (21%)	317/1261(25%)	31/48 (65%)	22/48 (46%)	371/856 (43%)
HR+/HER2−	8/21 (38%)	2/38 (5%)	30/463 (7%)	5/12 (42%)	10/30 (33%)	73/370 (20%)
HR+/HER2+	0/4 (0%)	1/6 (17%)	97/307 (32%)	0/2 (0)	0/4 (0)	101/167 (61%)
HR−/HER2+	0/0 (0%)	1/2 (50%)	109/174 (62%)	0/0 (0%)	1/1 (100%)	110/128 (85%)
TNBC	31/67 (46%)	11/27 (41%)	91/317 (29%)	26/34 (76%)	11/13 (85%)	87/191 (46%)

*pCR* pathologic complete response, *HR* hormone receptor, *HER2* human epidermal growth factor receptor 2, *TNBC* triple-negative breast cancer.

**Table 3:** Multivariable analysis of factors associated with pathologic complete response (pCR)

Characteristic	Univariate analysis			Multivariable analysis	
	No pCR *n* = 1045	pCR *n* = 381	*p*	Odds ratio (95% CI)	*p*
Age	50 (42–60)	51 (41–59)	0.9		
Race			0.8		
Black	149 (75%)	50 (25%)			
Asian, Native American, or other	174 (72%)	68 (28%)			
White	636 (73%)	239 (27%)			
Unknown	86	24			
Clinical T stage			0.028		
T1	148 (70%)	62 (30%)			
T2	565 (72%)	217 (28%)			
T3	228 (81%)	55 (19%)			
T4	99 (72%)	39 (28%)			
Unknown	5	8			
cN+	718 (75%)	234 (25%)	0.010		
*BRCA* status			0.001		
Noncarrier	934 (74%)	327 (26%)		ref.	ref.
* BRCA1*	53 (58%)	39 (42%)		2.37 (1.32–4.18)	0.003
* BRCA2*	58 (79%)	15 (21%)		1.56 (0.71–3.23)	0.2
Differentiation			<0.001		
Poor	660 (66%)	339 (34%)		ref.	ref.
Well/moderate	380 (91%)	37 (8.9%)		0.32 (0.19–0.52)	<0.001
Unknown	5	5			
Histology			<0.001		
Ductal	935 (73%)	351 (27%)			
Lobular/mixed	101 (87%)	15 (13%)			
Unknown	9	15			
Receptor subtype			<0.001		
HR+/HER2−	482 (92%)	40 (8%)		ref.	ref.
TN	278 (68%)	133 (32%)		3.95 (2.32–6.97)	<0.001
HR+/HER2+	219 (69%)	98 (31%)		6.16 (3.60–10.9)	<0.001
HR−/HER2+	66 (37.5%)	110 (62.5%)		21.6 (12.0–40.1)	<0.001
Neoadjuvant regimen			0.4		
ACT-based	969 (74%)	345 (26%)			
Taxane/platinum/CMF	74 (70%)	32 (30%)			
Unknown	2	4			
Carboplatin receipt	122 (67%)	60 (33%)	0.041		
Lymphovascular invasion	367 (88%)	48 (12%)	<0.001	0.48 (0.32–0.69)	<0.001
Unknown	181	149			

Categorical data presented as *n* (%) and continuous as median (interquartile range).

*CI* confidence interval, *HR* hormone receptor, *HER2* human epidermal growth factor receptor 2, *TN* triple negative, *ACT* adriamycin, cyclophosphamide, and taxol, *CMF* cyclophosphamide, methotrexate, and fluorouracil.

### Response to NAC in TN disease

Of the 67 *BRCA1* patients with TN disease, 31 (46%) experienced an overall pCR compared to 11/27 (41%) of *BRCA2* mutation carriers, and 91/317 (29%) of patients without a mutation. Younger age, *BRCA* status, and absence of LVI were associated with overall pCR on univariate analysis. In the adjusted analysis, age (OR 0.97, 95% CI 0.95, 1.00, *p* = 0.024) and LVI (OR 0.44, 95% CI 0.22, 0.83, *p* = 0.014) remained inversely associated with overall pCR but *BRCA* mutation status did not. Nodal pCR occurred in 26/34 (76%) of *BRCA1* carriers, 11/13 (85%) of *BRCA2* carriers, and 87/191 (46%) of noncarriers who presented with cN+ disease. Factors that were significantly associated with nodal pCR in TN disease included younger age (*p* < 0.001), Asian race (*p* = 0.026), *BRCA* mutation status (*p* < 0.001), and absence of LVI (*p* < 0.001). On multivariable analysis, younger age remained associated with nodal pCR (median [IQR] 52 [43, 60]) among patients without nodal pCR vs. 49 (36, 57) among patients with nodal pCR). LVI was less likely to be associated with pCR in this analysis (OR 0.29, 95% CI 0.13, 0.62; *p* = 0.002) (Table 4).

**Table 4:** Multivariable analysis of factors associated with overall pathologic complete response among TNBC patients

Characteristic	Univariate analysis			Multivariable analysis	
	No pCR *n* = 278	pCR *n* = 133	*p*	Odds ratio (95% CI)	*p*
Age	52 (44–60)	47 (36–56)	<0.001	0.97 (0.95, 1.00)	0.024
Race			0.12		
Black	61 (75%)	20 (25%)			
Asian, Native American, or other	42 (61%)	27 (39%)			
White	144 (64%)	80 (36%)			
Unknown	31	6			
Clinical T stage			0.2		
T1	33 (56%)	26 (44%)			
T2	165 (70%)	70 (30%)			
T3	60 (72%)	23 (28%)			
T4	19 (66%)	10 (34%)			
Unknown	1	4			
*BRCA* status			0.013		
Noncarrier	226 (71%)	91 (29%)		ref.	ref.
*BRCA1*	36 (54%)	31 (46%)		1.66 (0.81, 3.36)	0.2
*BRCA2*	16 (59%)	11 (41%)		1.97 (0.71–5.17)	0.2
Differentiation			0.062		
Poor	242 (66%)	124 (34%)			
Well/moderate	33 (80%)	8 (20%)			
Unknown	3	1			
Histology			0.07		
Ductal	264 (69%)	121 (31%)			
Lobular/mixed	13 (65%)	7 (35%)			
Unknown	1	5			
Neoadjuvant regimen			0.15		
ACT-based	263 (67%)	130 (33%)			
Taxane, platinum, or CMF	15 (83%)	3 (17%)			
Carboplatin	73 (68%)	37 (32%)	<0.001		
Lymphovascular invasion	81 (85%)	14 (15%)	<0.001	0.44 (0.22–0.83)	0.014
Unknown	52	56			

Categorical data presented as *n* (%) and continuous as median (interquartile range).

*TNBC* triple-negative breast cancer, *pCR* pathologic complete response, *CI* confidence interval, *ACT* adriamycin cyclophosphamide and taxol, *CMF* cyclophosphamide, methotrexate, and fluorouracil.

### Response to NAC in HR+/HER2− disease

Overall pCR occurred in 8/21 (38%) of *BRCA1* mutation carriers, 2/38 (5%) of *BRCA2* mutation carriers, and 30/463 (7%) of non-mutation carriers. Among patients with HR+/HER2-negative disease, *BRCA1* status (*p* < 0.001), poorly differentiated disease (*p* < 0.001), ductal histology (*p* = 0.012), HR-negative disease (*p* < 0.001), carboplatin use (*p* = 0.007), and absence of LVI (*p* < 0.001) were associated with pCR on univariate analysis. On multivariable analysis, *BRCA1* status remained significantly associated with pCR (Table 5). Compared to poorly differentiated disease, well-differentiated tumors were less likely to be associated with pCR (OR 0.05, 95% CI 0.01, 0.16; *p* < 0.001). Nodal pCR occurred in 5/12 (42%) of *BRCA1* mutation carriers, 10/30 (33%) of *BRCA2* mutation carriers, and 73/370 (20%) of non-mutation carriers. *BRCA* status (*p* = 0.040), poorly differentiated disease (<0.001), ductal histology (*p* = 0.022), carboplatin administration (*p* = 0.006), and absence of LVI (*p* < 0.001) were associated with nodal pCR on univariate analysis. On multivariable analysis, while *BRCA* status did not predict nodal pCR, well-differentiated disease, and LVI were inversely associated with nodal pCR.

**Table 5:** Multivariable analysis of factors associated with overall pathologic complete response among HR+/HER2− patients

Characteristic	Univariate analysis			Multivariable analysis	
	No pCR *n* = 482	pCR *n* = 40	*p*	Odds ratio (95% CI)	*p*
Age	50 (43–59)	50 (39–58)	0.5		
Race			>0.9		
Black	60 (92%)	5 (8%)			
Asian, Native American, or other	80 (92%)	7 (8%)			
White	308 (92%)	25 (8%)			
Unknown	34	3			
Clinical T stage			0.4		
T1	85 (93%)	6 (7%)			
T2	231 (91%)	24 (9%)			
T3	108 (96%)	5 (4%)			
T4	54 (93%)	4 (7%)			
Unknown	4	1			
*BRCA* status			<0.001		
Noncarrier	433 (94%)	30 (6%)		ref.	ref.
*BRCA1*	13 (62%)	8 (38%)		7.23 (2.49, 20.9)	<0.001
* BRCA2*	36 (95%)	2 (5%)		0.33 (0.02–1.66)	0.3
Differentiation			<0.001		
Poor	221 (86%)	37 (14%)		ref.	ref.
Well/moderate	259 (99%)	2 (1%)		0.05 (0.01, 0.16)	<0.001
Unknown	2	1			
Histology			0.012		
Ductal	409 (91%)	38 (9%)			
Lobular/mixed	68 (100%)	0 (0%)			
Unknown	5	2			
Neoadjuvant regimen			0.6		
ACT-based	465 (92%)	40 (8%)			
Taxane, platinum, or CMF	17 (100%)	0 (0%)			
Carboplatin	18 (75%)	6 (25%)	0.007		
Lymphovascular invasion	209 (98%)	5 (2%)	0.019		
Unknown	65	19			

Categorical data presented as *n* (%) and continuous as median (interquartile range).

*HR* hormone receptor, *HER2* human epidermal growth factor receptor 2, *pCR* pathologic complete response, *CI* confidence interval, *ACT* adriamycin cyclophosphamide and taxol, *CMF* cyclophosphamide, methotrexate, and fluorouracil.

## Discussion

This study compares rates of pCR between *BRCA1* and *BRCA2* variant carriers and noncarriers in, to our knowledge, one of the largest cohorts analyzed in this manner to date. Both overall pCR and nodal pCR more frequently occurred in *BRCA1* carriers compared to *BRCA2* carriers and noncarriers. When stratified by receptor subtype, we observed higher nodal pCR rates among *BRCA1* and *BRCA2* carriers compared to noncarriers within both subtype cohorts, including sufficient patients, namely HR+/HER2− and TNBC. In sensitivity analyses by subtype, *BRCA1* status was significantly associated with overall pCR among patients with HR+/HER2− disease.

The characteristics of *BRCA-*associated tumors were consistent with those in existing literature^4^. While 75% of *BRCA2-*associated breast cancers are reported to be HR+, approximately 70% of *BRCA1-*associated breast cancers are TNBC^20–23^. In this study, a similar rate of HR+ tumors was seen in *BRCA2* carriers and noncarriers, and *BRCA1* carriers more frequently had TNBC compared to *BRCA2* carriers and those with sporadic disease. Poorly differentiated tumors were more common in patients with *BRCA1* mutations. This is in line with reports that 66–100% of tumors in *BRCA1* mutation carriers exhibit grade 3 histology compared to 16–57% and 15–55% of disease in *BRCA2* carriers and sporadic breast cancers, respectively^24^.

Our finding that *BRCA1* carriers had a higher rate of pCR compared to *BRCA2* carriers and noncarriers supports previous studies that have assessed response to NAC in *BRCA1/2* mutation carriers. Arun et al. demonstrated higher odds of pCR in *BRCA1* carriers (26/57; 46%) than *BRCA*2 carriers (3/23; 13%) and noncarriers (53/237; 22%)^14^. In another study, Wunderle et al. observed that among patients treated with anthracycline-based NAC, pCR occurred more frequently in *BRCA1/2* carriers (11/25 [44%] *BRCA1* carriers and 4/13 [30%] *BRCA2* carriers) compared to noncarriers (30/230 [13%])^18^. When stratified by subtype, the proportion of patients with luminal A/B tumors who achieved pCR was highest among *BRCA1* carriers. In patients with TNBC, pCR rates were higher in *BRCA1/2* carriers compared to noncarriers. Together, these studies and ours corroborate preclinical data indicating differential response to chemotherapy by mutation status^3^. We were not, however, able to appreciate an association between *BRCA* status and overall pCR for TNBC or nodal pCR for TNBC or HR+/HER2− subtypes in adjusted sensitivity analysis. Although we postulate that this may have been due to limited sample size, our observation that *BRCA1* mutation status was significantly associated with overall pCR among those with HR+/HER2− disease after adjusting for clinically relevant factors merits further investigation.

Patients in this study uniformly received ACT. Addition of carboplatin was more common among *BRCA1/2* carriers compared to noncarriers. Many studies have demonstrated increased pCR rates with the addition of platinum to ACT in TNBC^25–27^. While carboplatin was significantly associated with pCR on univariate analysis, this significance dissipated after adjusting for other factors, likely due to sample size and the small number of pCR events, and was not included in our multivariable model. However, in this study, we observed that *BRCA1* patients with TNBC who received carboplatin had a pCR rate higher than that reported in existing literature for the general population^21–23^. These data are discordant with published literature from the subgroup analysis of the BrighTNess and GeparSixto trials^27,28^. These trials demonstrated that although pCR rates were increased among patients with TNBC who received neoadjuvant carboplatin in addition to ACT, pCR rates were not enhanced among those with germline *BRCA1/2* mutations compared to those without *BRCA1/2* mutations. These conflicting results may have been due to differences in sample size and analytic strategy. Compared to the GeparSixto and BrighTNess trials, we included a larger cohort of mutation carriers and analyzed data from *BRCA1* and *BRCA2* carriers separately. Future investigations are needed to determine how changes to standard NAC regimens will contribute to differences in pCR between *BRCA1/2* carriers and noncarriers, especially in those with TNBC who now receive chemoimmunotherapy given the findings of KEYNOTE-522^29^.

NAC chemotherapy importantly provides prognostic data and informs adjuvant treatment recommendations that improve overall survival. NAC is also frequently used to downstage locally advanced disease for the purposes of surgical de-escalation^30^. However, women with *BRCA1/2* mutations may prefer bilateral mastectomy for risk reduction, regardless of eligibility for breast conservation^31,32^. Accordingly, 90% of *BRCA1/2* carriers in this study underwent bilateral mastectomy. While management of in-breast disease may not be altered by NAC in mutation carriers, axillary downstaging after NAC remains a consideration. We report that nearly two-thirds of *BRCA1* carriers who had cN+ disease prior to NAC achieved pCR in their axilla. The nodal pCR rate among *BRCA1* carriers with HR+/HER2− disease was significantly higher than that reported in the literature for cases of sporadic cancer (7.5%–16.2%)^33^. As changes in the surgical management of the axilla during the time period of this study have made a larger proportion of women eligible for less invasive approaches, especially in HR+/HER2− disease, further study is needed to assess how germline genetics affect rates of axillary downstaging.

This study has several limitations. The small sample size, particularly for patients with HER2+ disease, precluded further subgroup analysis by receptor status. Additionally, where subset analysis was performed for TN and HR+/HER2− subtypes, a small sample size limits the ability for granular analysis. This study includes patients with cN+ disease who received NAC based on previously accepted guidelines and for whom systemic chemotherapy may no longer be the standard of care based on the RxPONDER trial^34^. Although a small proportion of patients may have received preoperative chemoimmunotherapy according to the KEYNOTE-522 regimen^35^, which became incorporated into our institutional practice in August 2021, we were not able to specifically account for the role of immunotherapy in our analysis of pCR. Additionally, we cannot account for bias that may have resulted in variability from clinician practices regarding patient selection for neoadjuvant treatment. In this study, patients were classified as noncarriers if they had negative genetic testing or did not meet the criteria for genetic testing based on NCCN guidelines; inaccurate assessment of mutational status may have influenced findings. We could not examine the association of pCR with survival because of short follow-up. As the majority of patients received ACT-based chemotherapy, our data provide little insight into treatment response to alternative regimens. Although our findings are consistent with existing literature, the single-institution nature of this study limits generalizability.

We conclude that *BRCA1*-associated breast cancer has a higher rate of overall and nodal pCR than both *BRCA2*-associated and sporadic disease. We demonstrate that pCR outcomes vary by *BRCA1* compared to *BRCA2* mutational status, and the importance of analyzing these groups separately in future studies. Lastly, these differences in pCR were observed in HR+/HER2− cancers that traditionally have lower response rates to NAC.

## Methods

### Study characteristics

After approval from the Memorial Sloan Kettering Cancer Center institutional review board, we identified consecutive women who were diagnosed with clinical stage I-III breast cancer and treated with NAC followed by surgery between November 2013 and January 2022. The need for informed consent was waived based on the retrospective nature of the study. Patients who qualified for genetic testing based on NCCN guidelines were referred for genetic counseling and evaluation for *BRCA1* and *BRCA2* germline variants^36^. Patients with *BRCA* variants of uncertain significance, non-*BRCA* deleterious pathogenic mutations, or missing pathology data were excluded. Genetic testing was either performed at our institution or outside the facility, and results were reviewed by the treating physicians.

Demographic, tumor, and treatment characteristics were abstracted from the electronic medical record. The tumor molecular subtype was defined as hormone receptor-positive (HR+) if ≥1% of tumor cells stained positive for estrogen receptor (ER) or progesterone receptor (PR) by immunohistochemistry^37^. HER2 overexpression was defined by immunohistochemistry or amplification based on fluorescence in situ hybridization according to ASCO/CAP guidelines^38^. In this study, clinically node-positive (cN+) patients were those who had evidence of malignant cells in ≥1 lymph node by fine needle aspiration or core needle biopsy at the time of diagnosis. PCR was defined as the absence of residual invasive tumor in the breast and ipsilateral axillary lymph nodes (ypT0/is ypN0).

Baseline disease characteristics were compared between *BRCA1* and *BRCA2* carriers and noncarriers using the Kruskal–Wallis rank sum test for continuous variables, and Fisher’s exact test or chi-square test for categorical variables. Logistic regression was used to evaluate the association between *BRCA1* or *BRCA2* mutation and pCR (i.e., ypT0/is pN0), adjusting for variables selected using backward elimination. *BRCA* status, clinical stage, differentiation, tumor subtype, NAC regimen, histology, and LVI were chosen a priori as clinically relevant variables and included for consideration by backward elimination. Given that studies have demonstrated pCR differs by tumor molecular subtype^39^, sensitivity analyses were performed to evaluate factors associated with pCR by subtype except in HER2+ disease, given the small sample size of *BRCA1/2* patients with HER2+ tumors. All analyses were performed using R 4.2 with a two-sided type I error rate (*α*) set to 0.05.

We have complied with all relevant ethical regulations, including the Declaration of Helsinki.

## Data Availability

The datasets used and/or analyzed during the current study are available from the corresponding author upon reasonable request.
